# Total metabolic lesion volume of lymph nodes measured by ^18^F-FDG PET/CT: a new predictor of macrophage activation syndrome in adult-onset Still’s disease

**DOI:** 10.1186/s13075-021-02482-2

**Published:** 2021-03-30

**Authors:** Liyan Wan, Yuting Gao, Jieyu Gu, Huihui Chi, Zhihong Wang, Qiongyi Hu, Jinchao Jia, Tingting Liu, Biao Li, Jialin Teng, Honglei Liu, Xiaobing Cheng, Junna Ye, Yutong Su, Chengde Yang, Hui Shi, Min Zhang

**Affiliations:** 1 Department of Rheumatology and Immunology, Ruijin Hospital, Shanghai Jiao Tong University School of Medicine, Shanghai, 200025 China; 2 Department of Nuclear Medicine, Ruijin Hospital, Shanghai Jiao Tong University School of Medicine, 197 Ruijin 2nd Road, Shanghai, 200025 China

**Keywords:** Adult-onset Still’s disease, Positron emission tomography/computed tomography, Disease severity, Macrophage activation syndrome

## Abstract

**Background:**

To investigate the potential utility of quantitative parameters obtained by ^18^F-fluorodeoxyglucose positron emission tomography/computed tomography (^18^F-FDG PET/CT) in the assessment of disease severity and the occurrence of macrophage activation syndrome (MAS) in adult-onset Still’s disease (AOSD).

**Methods:**

Fifty-seven patients with AOSD who underwent pre-treatment ^18^F-FDG PET/CT were recruited in this study and compared with 60 age- and sex-matched healthy controls. Clinical features and laboratory data were recorded. The systemic score was assessed to determine the disease severity. The maximal standardized uptake value (SUV_max_), metabolic lesion volume (MLV), and total lesion glycolysis (TLG) were used to evaluate the involved organs and tissues that abnormally accumulated ^18^F-FDG. Multivariate analysis was performed to identify the PET/CT-derived risk factors contributing to the AOSD-related MAS, and their diagnostic efficiency was evaluated.

**Results:**

High ^18^F-FDG accumulation was observed in the bone marrow (SUV_max_ median, 5.10), spleen (SUV_max_ median, 3.70), and lymph nodes (LNs, SUV_max_ median, 5.55). The SUV_max_ of the bone marrow (rho = 0.376, *p* = 0.004), SUV_max_ of the spleen (rho = 0.450, *p* < 0.001), TLG_total_ of LNs (rho = 0.386, *p* = 0.017), and MLV_total_ of LNs (rho = 0.391, *p* = 0.015) were correlated with the systemic score. The SUV_max_ of the spleen (*p* = 0.017), TLG_total_ of LNs (*p* = 0.045), and MLV_total_ of LNs (*p* = 0.012) were higher in patients with MAS than in those without MAS. A MLV_total_ of LNs > 62.2 (OR 27.375, *p* = 0.042) was an independent predictive factor for MAS with a sensitivity of 80.0% and a specificity of 93.9%.

**Conclusions:**

The glucose metabolic level of the spleen could be an effective and easy-to-use imaging indicator of disease severity, and MLV_total_ of LNs > 62.2 was a strong predictor of MAS occurrence in patients with AOSD.

## Introduction

Adult-onset Still’s disease (AOSD) is a systemic inflammatory disease characterized by spiking fever, salmon-pink evanescent rash, arthralgia, and hepatosplenomegaly [1]. The laboratory profile of AOSD is a reflection of systemic inflammation, including increased erythrocyte sedimentation rate (ESR) and C-reactive protein (CRP), as well as hyperferritinemia, leukocytosis, and elevated levels of cytokines [2]. Diagnosis of AOSD is based on clinical pattern recognition and exclusion of other autoimmune, inflammatory, infectious, or neoplastic diseases [3–5]. The patients with AOSD may experience life-threatening complications, especially companied with macrophage activation syndrome (MAS), with high mortality rate ranging from 10 to 42.5% [6, 7]. Early recognition and treatment of MAS is crucial but challenging, due to the lack of specific markers [8, 9].

^18^F-fluorodesoxyglucose positron emission tomography/computed tomography (^18^F-FDG PET/CT), which reflects both the glucose metabolic activity and the anatomical structure of target tissues, is commonly used on cancer diagnosis, staging, and prognosis, assessment of treatment response [10]. For AOSD, ^18^F-FDG PET/CT has been used as a whole-body imaging tool to exclude malignancies and identify the biopsy sites when the patients were at admission [11, 12]. Previous studies (13 cases [13], seven cases [14], and 26 cases [12]) have revealed the significant increase of FDG uptake of involved organs in patients with AOSD. However, the correlations between abnormal glucose metabolism of involved organs, as determined by the maximal standardized uptake value (SUV_max_), and disease severity are still illusive.

PET/CT-derived quantitative parameters based on three-dimensional volume of interest (VOI), namely metabolic tumor volume (MTV) and total lesion glycolysis (TLG), have been widely used in the assessment of whole-body disease burden and prognosis of tumors [15, 16]. Interestingly, recent studies presented associations between TLG and systemic inflammation in patients with recurrent colorectal cancer [17] and IgG_4_-related disease [18]. Here, we quantified ^18^F-FDG uptake in patients with AOSD using SUV, MTV, and TLG, in pursuit of identifying markers associated with disease severity and AOSD-related MAS as well.

## Material and methods

### Patient recruitment

From November 2015 to January 2019, we identified 57 hospitalized patients diagnosed with AOSD according to the classification criteria proposed by Yamaguchi et al. at an academic hospital [4]. Clinical manifestations and laboratory data were obtained on admission, and a systemic score, as proposed by Pouchot [19], was assessed for each patient. Macrophage activation syndrome (MAS) was diagnosed based on HLH-2004 criteria [20]. All patients underwent ^18^F-FDG PET/CT scan for excluding malignancy before treatments of AOSD. In addition, to determine the glucose metabolic level of normal tissues and organs on PET/CT, 60 age- and sex-matched healthy controls without a history of autoimmune disease, recent infection, or malignant tumor were retrospectively recruited. The study was approved by the Ethics Committee of Ruijin Hospital, Shanghai Jiao Tong University School of Medicine.

### PET/CT scanning

^18^F-FDG PET/CT scanning was performed using the Discovery VCT64 system (GE Healthcare, Chicago, IL, USA). To control the blood glucose concentration below 7.4 mmol/L before scanning, patients were instructed to fast for at least 6 h. Patients received an intravenous injection of 5–6 MBq of ^18^F-FDG per kilogram of body weight 45–60 min before scanning from the skull base to the mid-thigh. PET images were acquired for 3 min per bed position using a matrix size of 128 × 128, 28 subsets, two iterations, and full-width half-maximum post-filtering. CT images were acquired using a tube voltage of 140 kV, a tube current of 220 mA, and a section thickness of 3.75 mm. The reconstruction of PET images was based on an ordered-subset expectation maximization algorithm with photon attenuation correction using CT data.

### PET/CT image interpretation

Integrated PET and CT images were independently interpreted by two nuclear medicine physicians on the Advantage Workstation 4.4 system (GE Healthcare). The glucose metabolic level of the bone marrow, spleen, liver, and lymph nodes (LNs) in all patients with AOSD were assessed by the SUV_max_ and SUV_mean_ within the VOI drawn on the PET/CT images. The abnormal hypermetabolism of these organs and tissues were defined as SUV_max_ higher than the upper limit of the 95% confidence interval for the glucose metabolic level of them in the healthy controls. LNs with ^18^F-FDG uptake lower than blood pool were excluded from analyses. In this study, the MTV was renamed as the metabolic lesion volume (MLV) because AOSD-affected lesions were non-neoplastic. Both the MLV and TLG [18] were measured on all hypermetabolic LNs. The MLV was automatically delineated using a threshold of 40% of the SUV_max_. The TLG was defined as the MLV multiplied by the SUV_mean_. The total MLV (MLV_total_), which summed MTVs, and the total TLG (TLG_total_), which summed TLGs, in all hypermetabolic LNs were measured as the quantitative parameters of whole-body disease burden. Because of the irregular shape of the bone marrow and spleen, and the vicinity of the physiologically ^18^F-FDG-avid kidney, it was difficult to obtain VOIs completely covering either organ where the MLV and TLG could not be measured.

### Statistical analysis

Statistical analysis was performed using SPSS (version 23, IBM Corporation) and R (version 3.6.2) software packages. Chi-square and Fisher’s exact tests were used to compare categorical variables. The Mann-Whitney rank sum test was used to compare continuous variables with non-normal distribution after the Kolmogorov-Smirnov test. The Wilcoxon signed-rank test was used to compare paired continuous variables with non-normal distribution. Spearman’s correlations were analyzed to examine the relationship between PET/CT parameters and laboratory indices with a Bonferroni correction. Binary logistic regression and Firth logistic regression [21] were used to evaluate the contribution of different clinical risk factors to disease severity. All the variables were included in the regression mode. The variables with *p* < 0.1 in univariate analysis were entered in multivariate analysis. Multicollinearity was excluded from the final model. Receiver operating characteristic curves (ROCs) were performed to determine the diagnostic performance of the PET/CT parameters. The optimum cutoff value was defined based on the maximum Youden index. The diagnostic accuracy of each parameter was reported together with its 95% confidence interval (CI). Statistical significance was defined as *p* < 0.05.

## Results

### Clinical information of patients with AOSD

All the information is presented in Table 1. The median age of the 57 patients was 34, with a female predominance of 84.21%. The most prevalent clinical manifestations were arthralgia (91.23%) and skin rash (91.23%), followed by fever higher than 39 °C (80.70%). Increased levels of CRP (94.74%), serum ferritin (87.72%), and ESR (73.68%) were commonly observed. The median systemic score was 7.

**Table 1:** Patient characteristics

**Demographics**	
Number	57
Age (y)	34 (26–47)
Female	48/9
Disease duration (months)	3 (1–12)
**Clinical features**	
Fever > 39 °C	46 (80.7)
Skin rash	52 (91.2)
Arthralgia	52 (91.2)
Sore throat	44 (77.2)
Lymphadenopathy	43 (75.4)
Splenomegaly	25 (43.9)
Hepatomegaly	9 (15.8)
Pleuritis	20 (35.1)
Pericarditis	14 (24.6)
Weight loss	14 (24.6)
Lung disease	1 (1.8)
**Laboratory findings**	
Leukocytosis (> 10,000/mm^3^)	34 (59.7)
Neutrophils (> 80%)	36 (63.2)
Hb < 90 g/L	9 (15.8)
PLT < 100,000/mm^3^	2 (3.5)
ESR > 40 mm/h	42 (73.7)
CRP > ULN	54 (94.7)
Serum ferritin > 5 ULN	36 (63.2)
Abnormal liver function	36 (63.2)
Negative ANA (titer ≤ 1:80)	54 (94.7)
Negative RF	56 (98.2)
IL2R > ULN	33/45 (73.3)
Pouchot score	7 (5–7.5)

Data are presented in the form of median (Q1–Q3) as continuous variable or as absolute number (percentage of the total patients%);

*ULN* upper limit of normal, *Hb* hemoglobin, *PLT* platelet, *ESR* erythrocyte sedimentation rate, *ANA* antinuclear antibody, *RF* rheumatoid factor, *CRP* C-reactive protein, *IL-2R* interleukin-2 receptor

### Characterization of abnormal ^18^F-FDG accumulation in patients with AOSD

**Fig. 1:**
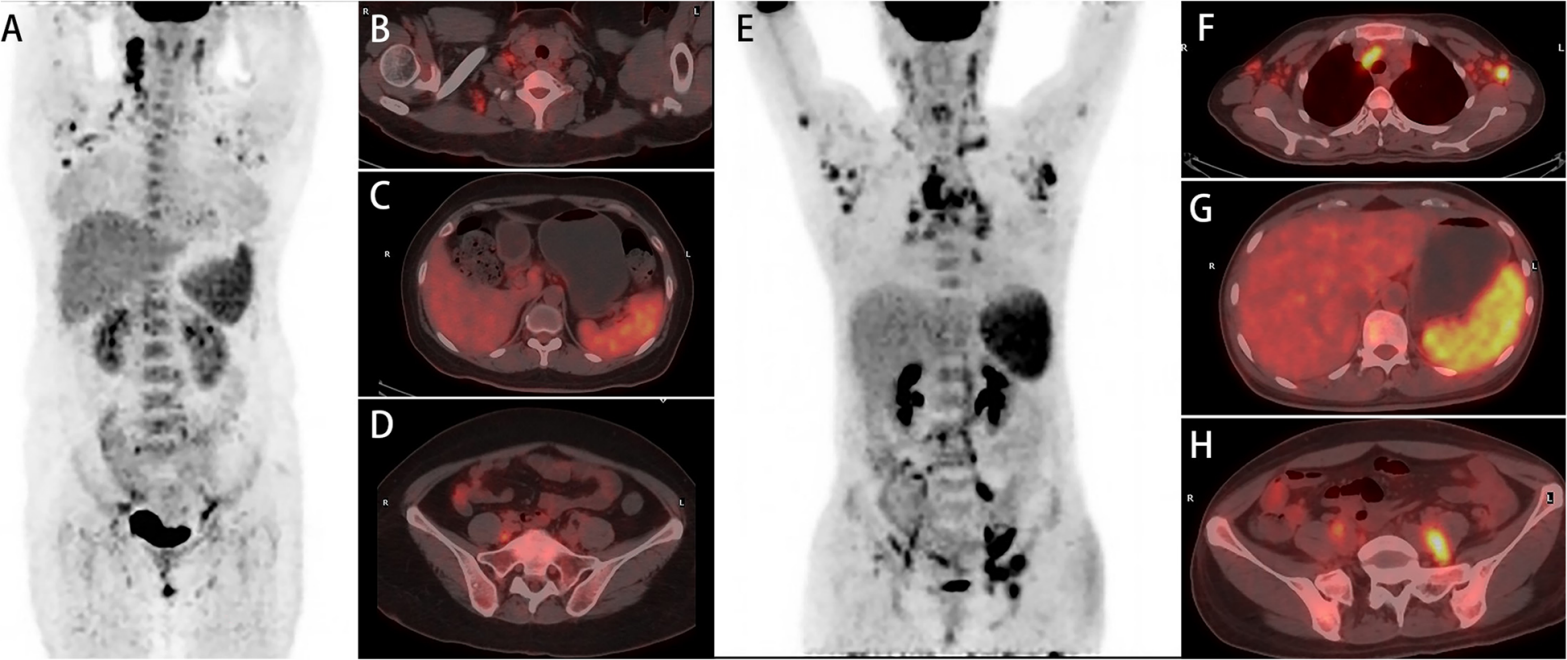
Whole-body PET image (**a**) and axial hybrid PET/CT images of the axillary LNs (**b**), liver and spleen (**c**), and pelvis bone (**d**) from a 45-year-old female patient with fever, skin rash, and arthralgia showed increased ^18^F-FDG uptake by the right axillary LNs (SUV_max_, 6.7), spleen (SUV_max_, 4.7), liver (SUV_max_, 3.3), and pelvic bone marrow (SUV_max_, 4.3). The TLG_total_ of LNs was 199,697.4, and MLV_total_ of LNs was 53.88. The patient did not progress to MAS after glucocorticoids and methotrexate treatment. Another 42-year-old female patient with fever, skin rash, and lymphadenopathy showed increased ^18^F-FDG uptake by the left axillary LNs (SUV_max_, 9.8), mediastinum LNs (SUV_max_, 11.4), spleen (SUV_max_, 7.5), liver (SUV_max_, 4.7), and pelvic bone marrow (SUV_max_, 5.1) on whole-body PET image (**e**) and axial hybrid PET/CT images of the axillary and mediastinum LNs (**f**), liver and spleen (**g**), and pelvis bone (**h**). The TLG_total_ of LNs was 1,144,469.8, and MLV_total_ of LNs was 244.75. The patient progressed to MAS after glucocorticoid treatment. Lately, she received intravenous immunoglobulins and achieved remission. LNs, lymph nodes; SUV, standardized uptake value; TLG, total lesion glycolysis; MLV, metabolic lesion volume; MAS, macrophage activation syndrome

The accumulation of ^18^F-FDG was significantly higher in the bone marrow, spleen, and LNs in patients with AOSD than healthy controls, except for ^18^F-FDG uptake in the liver (*p* = 0.672) (Table 2). Representative PET/CT images of abnormal hypermetabolic distributions in two patients with AOSD are depicted in Fig. 1.

**Table 2:** ^18^F-FDG accumulation in the mainly involved organs and tissues in patients with AOSD compared with healthy controls

	*N* (%)	SUV_max_ in patients with AOSD	Bilateral (*n*)	SUV_max_ in healthy controls	*p*
Bone marrow	–	5.10 (4.25–6.10)	–	3.76 (3.00–4.15)	< 0.001
Spleen	–	3.70 (3.05–4.35)	–	2.28 (2.06–2.56)	< 0.001
Liver	–	3.20 (2.80–3.60)	–	3.26 (2.88–3.58)	0.672
Lymph nodes^#^	38 (66.67)	5.55 (4.05–8.68)	–	–	–
Cervical LN	30 (52.63)	5.15 (3.92–7.85)	26/30	–	–
Axillary LN	27 (47.37)	4.70 (2.70–6.00)	25/27	–	–
Mediastinum LN	19 (33.33)	5.30 (3.70–8.90)	–	–	–
Pelvic LN	16 (28.07)	6.45 (4.68–8.52)	–	–	–
Retroperitoneal LN	14 (24.56)	5.60 (3.15–7.72)	–	–	–
Inguinal LN	13 (22.81)	4.20 (2.65–5.20)	10/13	–	–
Abdominal LN	8 (14.04)	6.60 (5.25–7.70)	–	–	–

Data are presented in the form of median (Q1–Q3) as continuous variable

*SUV* standardized uptake value

^#^This was calculated from the patients with hypermetabolic lymph nodes (*n* = 38). SUVmax was the maximum SUV of the regional lymph node

Bone marrow hypermetabolism occurred in 61.40% (35/57) of the patients, while spleen hypermetabolism occurred in 75.44% (43/57) of the patients. 66.7% (38/57) patients showed hypermetabolic LNs. In these cases, bilateral distribution of hypermetabolic LNs was commonly observed. The most commonly involved superficial LNs were cervical LNs (30/38, 78.95%) and axillary LNs (27/38, 71.05%), followed by inguinal LNs (13/38, 34.21%). Deep hypermetabolic LNs were also frequently observed, ranking with mediastinum LNs (19/38, 50.00%), pelvic LNs (16/38, 42.11%), retroperitoneal LNs (14/38, 36.84%), and abdominal LNs (8/38, 21.05%). In addition, four patients exhibited hypermetabolic LNs at the unilateral upper arm (intra-muscle).

Because of the limited scan range of PET/CT in this study, only the hip and shoulder joints were evaluated. Increased ^18^F-FDG uptake by the hip joints was only seen in one patient, with a SUV_max_ of 3.1, whereas three patients showed hypermetabolic shoulder joints with SUV_max_ of 3.1–3.3.

Abnormal FDG uptake by the right subscapularis was observed in only one patient. There was no increased ^18^F-FDG uptake by the skin in our cohort. In addition, there was only one (1.75%) patient in our cohort who showed parenchymal lung involvement with a SUV_max_ of 2.2 on the lung.

### Correlations between laboratory findings, systemic score, and PET/CT parameters

The correlations between disease severity-related laboratory findings and PET/CT parameters are summarized in Table S1. The spleen hypermetabolism showed positive correlations with ferritin (rho = 0.478, *p* < 0.001), lactate dehydrogenase (LDH) (rho = 0.462, *p* < 0.001), and interleukin-2 receptor (IL-2R) (rho = 0.454, *p* = 0.002). The SUV_max_ of the LN negatively correlated with the white blood cell count (rho = − 0.500, *p* = 0.001). The MLV_total_ of LNs displayed a positive correlation with the levels of IL-2R (rho = 0.495, *p* = 0.004).

**Fig. 2:**
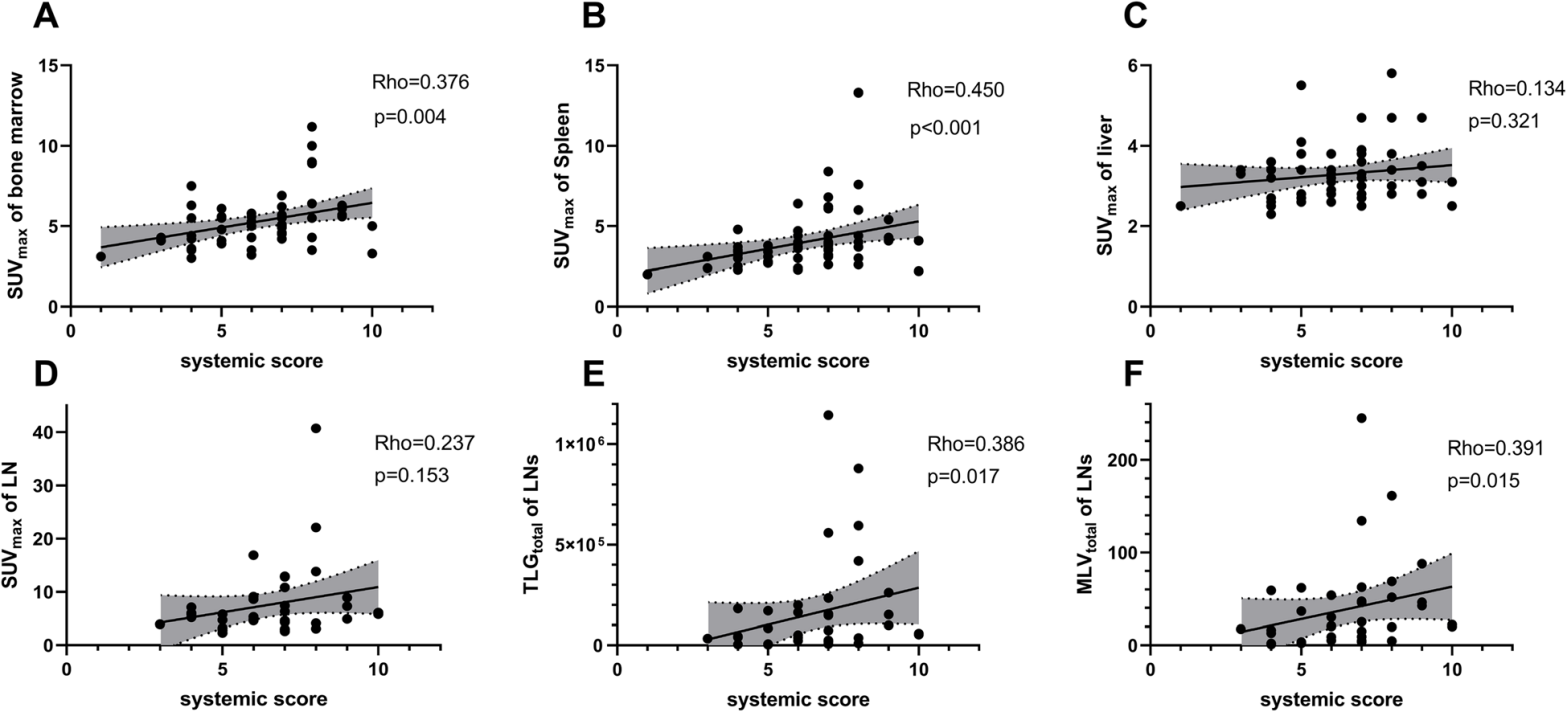
Correlations between the SUV_max_ of the bone marrow (**a**), spleen (**b**), liver (**c**), LNs (**d**), TLG_total_ (**e**), MLV_total_, (**f**) of LNs, and the systemic score. SUV, standardized uptake value; TLG, total lesion glycolysis; MLV, metabolic lesion volume. Significance was set at *p* < 0.05

The systemic score is a widely used indicator of AOSD disease severity. The SUV_max_ of the spleen (rho = 0.450, *p* < 0.001), SUV_max_ of the bone marrow (rho = 0.376, *p* = 0.004), TLG_total_ (rho = 0.386, *p* = 0.017), and MLV_total_ (rho = 0.391, *p* = 0.015) of LNs showed significant correlations with the systemic score (Fig. 2). Notably, the patients with systemic score ≥ 7 showed significantly higher SUV_max_ of the bone marrow (*p* = 0.005), spleen (*p* = 0.002), TLG_total_ (*p* = 0.044), and MLV_total_ (*p* = 0.033) of LNs than those with systemic score < 7 (Table S2).

### PET/CT features in MAS

Eight of 57 patients were diagnosed with MAS during the current hospitalization and underwent PET/CT scanning before the diagnosis of MAS. The time interval between PET/CT scanning and the diagnosis of MAS ranged from 2 to 33 days (Table S3). Only two patients showed cytopenia involving cells of 1 lineage, suggesting MAS might be in development when performing PET/CT.

Patients with MAS showed significantly higher SUV_max_ of the spleen (*p* = 0.017, *n* = 8), TLG_total_ (*p* = 0.045, *n* = 5), and MLV_total_ of LNs (*p* = 0.012, *n* = 5) compared to patients without MAS (Table 3). Considering the high mortality rate of MAS, the risk factors for MAS were assessed (Table 4). ROC analyses revealed that MLV_total_ of LNs > 62.2, SUV_max_ of the spleen > 3.55, and TLG_total_ of LNs > 248,040 were the optimal cut-points for discriminating MAS occurrence. Elevated levels of LDH, IL-2R, platelet count, TLG_total_ of LNs > 248,040, SUV_max_ of the spleen > 3.55, and MLV_total_ of LNs > 62.2 significantly associated with MAS occurrence in univariate analysis. Subsequent multivariate analyses indicated that elevated IL-2R level and MLV_total_ of LNs > 62.2 were independent risk factors of MAS.

**Table 3:** Comparison of PET/CT parameters between the patients with and without MAS

	Patients with MAS	Patients without MAS	*p*
	*n* = 8	*n* = 49	
SUV_max_ of the bone marrow	4.95 (3.68–5.40)	5.50 (4.30–6.15)	0.214
SUV_max_ of the spleen	4.35 (3.78–5.75)	3.50 (3.00–4.10)	0.017
SUV_max_ of the liver	3.10 (2.85–3.65)	3.30 (2.75–3.60)	0.854
SUV_max_ of LNs	7.30 (4.65–11.80)	5.20 (4.00–8.00)	0.271
TLG_total_ of LNs	261,170.20 (86,548.35–851,990.60)	44,518.30 (8828.20–159,463.70)	0.045
MLV_total_ of LNs	87.81 (38.78–189.47)	20.05 (4.74–46.12)	0.012

Data are presented in the form of median (Q1–Q3) as continuous variable

**Table 4:** Risk factors for the occurrence of MAS

	Univariate analysis			Multivariate analysis			
	*p*	OR	95% CI	*p*	OR	95% CI	
Age	0.138	0.955	0.898–1.015				
Sex	0.784	1.366	0.147–12.677				
LDH	0.009	1.005	1.001–1.008				
PLT	0.035	0.992	0.984–0.999				
IL-2R	0.002	1.002	1.001–1.003	0.002	1.003	1.001–1.007	
SUV_max_ of the spleen > 3.55	0.004	19.170	2.182–2528.558				
TLG_total_ of LNs > 248,040	0.013	15.000	1.752–128.391				
MLV_total_ of LNs > 62.2	0.002	62.000	4.529–848.704	0.042	27.375	1.117–123,080.200	

Both TLG_total_ and MLV_total_ of LNs were calculated from the patients with hypermetabolic lymph nodes (*n* = 38). All laboratory variables were included in logistic regression, and only the results with *p* < 0.1 were listed here

*LDH* lactate dehydrogenase, *PLT* platelet, *IL-2R* interleukin-2 receptor, *SUV* standardized uptake value, *LN* lymph node, *TLG* total lesion glycolysis, *MLV* metabolic lesion volume, *MAS* macrophage activation syndrome

MLV_total_ of LNs > 62.2 had a sensitivity of 80.0%, a specificity of 93.9%, and an AUC of 0.855 in predicting MAS occurrence, which were comparable with those of the traditional marker, IL-2R (sensitivity, 75.0%; specificity, 97.3%; AUC, 0.899). Level of ferritin, SUV_max_ of the spleen, and the systemic score showed low specificity of 40.8–55.1% and AUC of 0.642–0.765 (Table 5).

**Table 5:** The AUC, sensitivity, specificity, and likelihood ratio of PET/CT parameters, serologic markers, and systemic scoring for predicting the occurrence of MAS

Factor	AUC (95% CI)	Cutoff value	Sensitivity, % (95% CI)	Specificity, % (95% CI)	Likelihood ratio
IL-2R	0.899 (0.776–0.999)	2824 pg/ml	75.0 (40.9–95.6)	97.3 (48.7–99.9)	27.8
MLV_total_ of LNs	0.855 (0.642–0.999)	62.2	80.0 (37.6–99.0)	93.9 (80.4–98.9)	13.2
SUV_max_ of the spleen	0.765 (0.630–0.900)	3.55	100.0 (67.6–100.0)	53.1 (39.4–66.3)	2.13
Systemic score ≥ 7	0.713 (0.541–0.885)		87.5 (52.9–99.4)	55.1 (41.3–68.1)	1.95
Ferritin > 5 ULN	0.642 (0.455–0.828)		87.5 (52.9–99.4)	40.8 (28.2–54.8)	1.48

*LN* lymph node, *MLV* metabolic lesion volume, *IL-2R* interleukin-2 receptor, *MAS* macrophage activation syndrome. MLV_total_ of LNs were calculated from the patients with hypermetabolic lymph nodes (*n* = 38)

## Discussion

^18^F-FDG PET/CT is a useful tool to rule out malignancy before the diagnosis of AOSD. The additional values including the potential correlations between the PET/CT findings and disease assessments are worth to be investigated. Hypermetabolic status of the bone marrow, spleen, and LNs in patients with AOSD have been observed in other studies [11–14, 22–25]. We further explored the utility of PET/CT to assess disease severity and MAS occurrence of AOSD. Our study suggests that PET/CT could act as an early detective tool to evaluate the disease severity and predict the occurrence of deadly complication, MAS, in patients with AOSD.

The glucose metabolic level of the spleen could be an effective indicator of AOSD severity. In the present study, not only positive correlations between SUV_max_ of the spleen and those laboratory profiles of AOSD including the levels of LDH, ferritin, and IL-2R were observed, but also the hypermetabolism of the spleen showed a higher correlation coefficient with systemic score and more frequent occurrence in patients with AOSD than those of the bone marrow and LNs. We assumed that the enhanced FDG accumulation in the spleen may be secondary to inflammation and hypercytokinemia [26]. Furthermore, compared to the bone marrow and LNs, the spleen as a morphologically oblate and single organ is more likely to be drawn in a VOI on PET/CT images for more reliably and easily measuring its glucose metabolic levels in clinical practice. Additionally, a negative correlation between SUV_max_ of LNs and the white blood cell count was observed, which might be due to virus infection which was known as a trigger to AOSD [27–29], thereby temporarily disrupting the homeostasis of the bone marrow [30]. Although both elevated [24] and reduced [31] ^18^F-FDG uptake of the liver was observed in previous case reports, there was no statistical difference of SUV_max_ of the liver between patients with AOSD and healthy controls in our study. Similar to the studies of Dong et al. [12] and Jiang et al. [11], the prevalence of articular involvement in our cohort (10.53%) was low, indicating that increased ^18^F-FDG uptake in the joints may not be significant in patients with AOSD, which was probably due to the relatively mild involvement of joints in Chinese patients [32].

MLV_total_ of LNs was a promising useful predictor of MAS occurrence in patients with AOSD. Previous studies have reported that ^18^F-FDG PET/CT-derived quantitative parameters were associated with the occurrence [33] or outcome of MAS [34, 35]. In our study, we further found that MLV_total_ of LNs had better diagnostic performance than SUV_max_ of the spleen and TLG_total_ of LNs for distinguishing patients with MAS from those without MAS. Hypermetabolism of whole-body LNs is believed to be due to the high accumulation of inflammatory cells and the over-activation of the mononuclear phagocyte system in LNs [34, 36]. Patients with MLV_total_ of LNs > 62.2 showed a 20-fold increased risk for MAS occurrence than those with MLV_total_ of LNs less than or equal to 62.2. IL-2R may serve as a good laboratory indicator of MAS [37]. In this study, the IL-2R level was also an independent factor for predicting MAS occurrence with comparably high likelihood ratios to MLV_total_ of LNs. Even if the predictive value of IL-2R, as a cheaper test than PET/CT, was already high, the value of PET/CT was not negligible. Firstly, the performance of IL-2R for diagnosing MAS is still limited [38, 39], especially with low specificity (38.8–72.5%). Secondly, PET/CT has been routinely used to rule out malignancy in the diagnosis of AOSD. Its evaluation of the MAS occurrence could bring additional diagnostic information to clinicians. We believe that the combined diagnosis of IL-2R and MLV_total_ of LNs may have better predictive performance for MAS, but this conclusion cannot be obtained due to the limited number of samples in the present study. Therefore, further attention should be given to the whole-body inflammatory burden reflected by the MLV_total_ of LNs for assessing the risk of MAS in patients with AOSD. Recently, the negative prognostic impact of lung involvement in patients with AOSD has been reported [40], and the lung involvement has been correlated with the occurrence of MAS [41, 42]. However, due to the low prevalence of parenchymal lung involvement in our study, no inferential analysis was made.

There are still some limitations in this study. (a) The number of cases with MAS was low (*n* = 8) leading to the possibility of model overfitting. (b) Short-term glucocorticoid treatment before PET/CT scanning in some patients may have a limited effect on the accuracy of SUV measurement [43]. (c) There was a short time interval between PET/CT and diagnosis of MAS in two patients, whereas other six patients were diagnosed with MAS 1 week after PET/CT scan. Therefore, PET/CT may act as an actual display of the current disease activity as well as a prediction over time. This study was a pilot study at a single center, and more high evidence studies are needed to verify the reliability of the PET/CT parameters in the assessment of disease severity and the prediction of MAS in patients with AOSD.

## Conclusions

Our data revealed that the glucose metabolic level of the spleen could be an effective and easy-to-use imaging indicator of disease severity, and MLV_total_ of LNs > 62.2 was a strong predictor of MAS occurrence in patients with AOSD.

## Supplementary Information

**Additional file 1: Table S1.** Correlations between disease severity-related laboratory findings, systemic score, and PET/CT parameters. **Table S2.** Comparison of PET/CT parameters between patients with systemic score ≥ 7 and patients with systemic score <7. **Table S3.** Characteristic of eight patients with MAS.

## Data Availability

All data generated or analyzed during this study are included in this published article and its supplementary information files.

## Abbreviations

^18^F-FDG PET/CT: ^18^F-fluorodeoxyglucose positron emission tomography/computed tomography
AOSD: Adult-onset Still’s disease
AUC: Area under the curve
CI: Confidence interval
CRP: C-reactive protein
csDMARDs: Conventional synthetic disease-modifying anti-rheumatic drugs
CT: Computer tomography
ESR: Erythrocyte sedimentation rate
IL: Interleukin
LNs: Lymph nodes
MAS: Macrophage activation syndrome
MLV_total_: Total metabolic lesion volume
MRI: Magnetic resonance imaging
VOI: Volume of interest
MTX: Methotrexate
NSAIDs: Nonsteroidal anti-inflammatory drugs
SUV_max_: Maximal standardized uptake value
MTV: Metabolic tumor volume
TLG: Total lesion glycolysis
TNF: Tumor necrosis factor
TLG_total_: Total lesion glycolysis
ROC: Receiver operating characteristic curve
