# Supplementary material for: Total metabolic lesion volume of lymph nodes measured by 18F-FDG PET/CT: a new predictor of macrophage activation syndrome in adult-onset Still’s disease
Source: Arthritis Res Ther. 2021 Mar 30;23:97. doi: 10.1186/s13075-021-02482-2 (PMC8008587; doi:10.1186/s13075-021-02482-2)
Supplement: Supplementary file 1 — Additional file 1: Table S1. Correlations between disease severity-related laboratory findings, systemic score, and PET/CT parameters. Table S2. Comparison of PET/CT parameters between patients with systemic score ≥ 7 and patients with systemic score <7. Table S3. Characteristic of eight patients with MAS. [file 13075_2021_2482_MOESM1_ESM.docx]

**Table S1.** **Correlations between disease severity-related laboratory findings, systemic score, and PET/CT parameters**

|  | SUVmax of BM | | SUVmax of spleen | | SUVmax of LN | | TLG_total_ of LNs | | MLV_total_ of LNs | |
| --- | --- | --- | --- | --- | --- | --- | --- | --- | --- | --- |
|  | Rho | p | Rho | p | Rho | p | Rho | p | Rho | p |
| WBC | 0.063 | 0.641 | -0.155 | 0.250 | -0.500 | 0.001 | -0.415 | 0.010 | -0.365 | 0.024 |
| CRP | 0.354 | 0.007 | -0.009 | 0.947 | 0.246 | 0.137 | 0.244 | 0.140 | 0.178 | 0.285 |
| ESR | 0.123 | 0.361 | 0.013 | 0.922 | -0.054 | 0.746 | -0.145 | 0.386 | -0.141 | 0.398 |
| Ferritin | 0.217 | 0.104 | 0.478 | <0.001 | 0.026 | 0.876 | 0.302 | 0.066 | 0.353 | 0.030 |
| LDH | 0.241 | 0.071 | 0.462 | <0.001 | 0.200 | 0.228 | 0.314 | 0.055 | 0.349 | 0.032 |
| IL-1β | 0.311 | 0.038 | -0.173 | 0.256 | -0.323 | 0.072 | -0.231 | 0.203 | -0.159 | 0.385 |
| IL-2R | -0.133 | 0.384 | 0.454 | 0.002 | 0.053 | 0.774 | 0.359 | 0.044 | 0.495 | 0.004 |
| IL-6 | 0.231 | 0.131 | -0.095 | 0.540 | -0.272 | 0.132 | -0.201 | 0.269 | -0.139 | 0.449 |
| TNF | 0.163 | 0.297 | 0.320 | 0.037 | 0.085 | 0.648 | 0.340 | 0.061 | 0.458 | 0.010 |

WBC, white blood cell; CRP, c-reactive protein; ESR, erythrocyte sedimentation rate; ALT, alanine aminotransferase; AST, aspartate aminotransferase; LDH, lactate dehydrogenase; IL-1β, interleukin-1β; IL-2R, interleukin-2 receptor; TNF, tumor necrosis factor; BM, bone marrow; LN, lymph node; SUV, standardized uptake value; TLG, total lesion glycolysis; MLV, metabolic lesion volume. SUV_max_ of LNs was the maximum SUV of the hypermetabolic lymph node in the whole body. SUV_max_, TLG_total_, and MLV_total_ were calculated from the patients with hypermetabolic lymph nodes (n=38).

**Table S2. Comparison of PET/CT parameters between patients with systemic score ≥ 7 and patients with systemic score <7**

|  | Systemic score ≥ 7 | Systemic score <7 |  |
| --- | --- | --- | --- |
|  | n=29 | n=28 | p |
| SUVmax of bone marrow | 5.60(4.75-6.25) | 4.35(3.68-5.60) | 0.005 |
| SUVmax of spleen | 4.00(3.35-5.70) | 3.15(2.73-3.78) | 0.002 |
| SUVmax of liver | 3.10(2.80-3.70) | 3.25(2.70-3.55) | 0.614 |
| SUVmax of lymph nodes | 6.10(3.60-11.80) | 5.20(4.25-6.55) | 0.428 |
| TLG of lymph nodes | 99520.20(21153.75-340088.5) | 38634.30(5241.35-124188.45) | 0.044 |
| MLV of lymph nodes | 42.61(12.14-65.72) | 16.14(2.89-33.50) | 0.033 |

SUV, standardized uptake value; TLG, total lesion glycolysis; MLV, metabolic lesion volume. SUV_max_ of LNs was the maximum SUV of the hypermetabolic lymph node in the whole body. SUV_max_, TLG_total_, and MLV_total_ of lymph nodes were calculated from the patients with hypermetabolic lymph nodes (n=38).

**Table S3. Characteristic of eight patients with MAS**

| Patient no. | 1 | 2 | 3 | 4 | 5 | 6 | 7 | 8 |
| --- | --- | --- | --- | --- | --- | --- | --- | --- |
| Age(y)/  sex | 21/F | 28/F | 27/F | 25/F | 18/F | 40/F | 33/M | 45/F |
| Clinical manifestations | fever, sore throat, arthralgia, hepatomegaly, splenomegaly | fever, rash, abnormal liver function, splenomegaly | fever, arthralgia, rash, sore throat, splenomegaly | fever, arthralgia, rash, sore throat, hepatomegaly, splenomegaly | fever, lymphadenopathy, arthralgia, splenomegaly | fever, rash, lymphadenopathy, arthralgia, splenomegaly | fever, arthralgia, abnormal liver function | fever, rash, arthralgia, hepatomegaly |
| N (*10^9/L) | 8.2 | 11.3 | 2.4 | 11.5 | 8.6 | 4.7 | 26.1 | 17.1 |
| Hb (g/dL) | 11.9 | 8.9 | 9.2 | 9.2 | 11.2 | 10.4 | 11.8 | 12.5 |
| PLT (*10^9/L) | 49 | 261 | 159 | 268 | 312 | 119 | 249 | 336 |
| LDH (IU/L) | 1792 | 592 | 730 | 1521 | 666 | 742 | 546 | 365 |
| AST (IU/L) | 3102 | 64 | 84 | 131 | 54 | 113 | 49 | 41 |
| ferritin ≥ 500μg/L | 1 | 1 | 1 | 1 | 1 | 1 | 1 | 0 |
| IL-2R (U/ml) | 6500 | 3903 | 3060 | 3020 | 2832 | 2829 | 1441 | 1196 |
| Treatment before the PET/CT scan | MP 40mg qd | MP 80mg qd, MTX 10mg qw, Thalidomide | MP 80mg qd, MTX 10mg qw | MP 80mg qd, HCQ 200mg bid | MP 80mg qd | antibiotics | MP 40mg qd | MP 80mg qd |
| Time interval | 2 days | 30 days | 2 days | 8 days | 31 days | 33 days | 25 days | 28 days |
| SUV_max_ of BM | 4.20 | 5.50 | 4.90 | 5.00 | 5.70 | 5.10 | 3.50 | 3.50 |
| SUV_max_ of spleen | 4.40 | 4.00 | 6.20 | 3.60 | 4.30 | 8.40 | 3.70 | 4.40 |
| SUV_max_ of liver | 3.20 | 3.00 | 2.50 | 3.00 | 2.80 | 4.70 | 3.20 | 3.80 |
| SUV_max_ of LN | 6.30 | 3.00 | 10.80 | \ | 7.30 | 12.80 | \ | \ |
| TLG of LNs | 148834.6 | 24262.10 | 559511.4 | \ | 261170.2 | 1144469.8 | \ | \ |
| MLV of LNs | 62.59 | 14.97 | 134.19 | \ | 87.81 | 244.75 | \ | \ |

F, female; M, male; N, Neutrophils; Hb, Hemoglobin; PLT, platelet; LDH, lactate dehydrogenase; IL-2R, interleukin-2 receptor; MP, Methylprednisolone; MTX, methotrexate; HCQ, Hydroxychloroquine; BM, bone marrow; SUV, standardized uptake value; LN, lymph node; TLG, total lesion glycolysis; MLV, metabolic lesion volume; MAS, macrophage activation syndrome. Both TLGtotal and MLVtotal of LNs were calculated from the patients with hypermetabolic lymph nodes (n=5).
